# The Overall Survival and Safety of Men with Metastatic Hormone-Sensitive Prostate Cancer Treated with Combination Therapy of Novel Androgen Receptor Antagonists and Androgen-Deprivation Therapy: A Systematic Review and Meta-Analysis

**DOI:** 10.1155/2022/6211059

**Published:** 2022-08-26

**Authors:** Xupeng Wu, Haisong Han, Chao Zhang, Wei Song

**Affiliations:** Department of Urology, Jincheng People's Hospital, Jincheng, China

## Abstract

**Background:**

Several novel androgen receptor antagonists have been introduced into the treatment of metastatic hormone-sensitive prostate cancer (mHSPC). We conducted a meta-analysis to evaluate the survival and safety of the combination therapy of novel androgen receptor antagonist and androgen-deprivation therapy (ADT) in patients with mHSPC.

**Methods:**

Electronic databases were searched for randomized controlled trials (RCTs) of ADT combined with novel androgen receptor antagonists compared with ADT alone in men with mHSPC. Revman 5.4 and STATA 14.0 were used to performed the meta-analysis, and hazard ratio (HR) and odds ratio (OR) with 95% confidence intervals (CIs) were used as the measurement indicators of outcome variables.

**Results:**

Six RCTs were eventually identified for meta-analysis. Compared with ADT alone, the combination therapy of novel androgen receptor antagonists and ADT can significantly improve the overall survival (OS) and progression-free survival (PFS), as the pooled HR were 0.66, 95%CI (0.60, 0.72), *P* < 0.00001 and 0.43, 95%CI (0.34, 0.54), *P* < 0.00001, respectively, despite increasing the risk of any serious adverse events (OR: 1.18, 95%CI (1.04, 1.33), *P*=0.008). *Discussion*. This study showed that compared with ADT alone, the combination therapy of novel androgen receptor antagonists and ADT can significantly improve the survival status of mHSPC patients, while it increases the risk of serious adverse events.

## 1. Introduction

Prostate cancer (PC) is one of the most common male malignant tumors and the momentous causes of cancer-related death in the world. Globally, PC accounts for about 15% of the new cancer cases in men and has become the second highest incidence of male cancer in the world after lung cancer [1].

A considerable number of PC patients have had distant metastasis at the time of treatment, among which bone metastasis is the most common. Although metastasis has occurred, the prognosis of metastatic PC is relatively better than that of other advanced tumors [2]. The main reason is that most newly diagnosed metastatic PC belongs to metastatic hormone sensitive prostate cancer (mHSPC). The activity of tumor cells in both primary and metastatic lesions depends on androgens, and endocrine therapy based on androgen deprivation therapy (ADT) is always needed [3, 4].

At present, ADT is the basic treatment for patients with mHSPC, which can control and alleviate the condition of most mHSPC and can obtain good clinical efficacy. Although its initial efficacy is significant, patients will develop metastatic castration resistant prostate cancer (mCRPC) after 18 months, and the median survival time is often less than 2 years [5]. Therefore, delaying the occurrence of mCRPC is expected to further improve the survival of mHSPC patients [6].

Since 2005, based on the in-depth study of the pathogenesis of mHSPC, a variety of new treatment strategies and drugs have significantly improved the prognosis of mHSPC patients, including docetaxel chemotherapy, new androgen synthesis inhibitors, novel androgen receptor antagonist, and targeted drugs for homologous recombination repair defects [7–10]. In recent years, novel androgen receptor antagonists have been used in some clinical trials in combination with ADT in the treatment of mHSPC and have achieved significant therapeutic effects. Their representative drugs include abiraterone, enzalutamide, apalutamide, and darolutamide [11–14]. Our study integrated the overall effects of a variety of novel androgen receptor antagonists and has compared the survival and safety of ADT combined with the above novel androgen receptor antagonist with that of ADT alone by meta-analysis, in order to help clinicians better understand the treatment and management of mHSPC.

## 2. Methods

### 2.1. Literature Search Strategy

The searchable databases include PubMed, EMBASE, Cochrane Library, and Web of Science, and two of the authors (X Wu and H Han) searched the articles published from January 2000 to May 2022 on the treatment of mHSPC by using a combination therapy of novel androgen receptor antagonists and ADT. The search was performed by the combination of the following search terms using the Boolean operators “OR” and/or “AND”: “metastatic prostate cancer,” “androgen-deprivation therapy,” “abiraterone,” “enzalutamide,” “apalutamide,” and “darolutamide.” A comprehensive search of the literature was performed without restrictions of publication language and publication status. In addition, other two authors (C Zhang and W Song) manually searched the references of the selected articles and the previous meta-analysis to screen the potential relevant citations. Detailed search strategies of the above databases are presented in Table 1.

### 2.2. Study Selection

Studies that meet the following PICOS principles were considered eligible for inclusion in the study: (P) patients with mHSPC, (I) intervention therapy: combination therapy of ADT and novel androgen receptor antagonists, such as abiraterone, enzalutamide, apalutamide, and darolutamide, (C) control therapy: ADT alone, (O) outcomes: survival outcomes, such as overall survival (OS) and progression-free survival (PFS), safety outcomes, such as any adverse event (AE), grade 3 or 4 AE, any serious AEs, and AEs leading to death, and (S) study design: we only included randomized controlled trials (RCTs) with the highest level of evidence quality.

### 2.3. Data Extraction and Quality Assessment

Two independent reviewers (X Wu and C Zhang) performed the data extraction and quality assessment, and the disagreements were resolved by consensus or by consulting a third reviewer (W Song). The following data were extracted: first author's name, year of publication, author's country, trail phase and name, intervention, number of patients assigned to each group, and participant characteristics. The subjective assessment of the included studies' methodological quality was performed by all the authors by using the Cochrane Collaboration tool.

### 2.4. Statistical Analysis

Revman (version 5.4, Cochrane Collaboration) and STATA (version 14.0, STATA Corporation) were used to estimate the overall pooled effect size for each outcome. The prespecified survival outcomes were OS and PFS, and the safety outcomes were different types of AEs. Survival outcomes were reported as a hazard ratio (HR) with 95% confidence intervals (CIs), and safety outcomes were reported as an odds ratio (OR) with 95% CIs. The chi square test and I^2^ statistics were used to test the heterogeneity. It was generally believed that I^2^ ≥ 50% indicated substantial heterogeneity. For homogeneous studies, the fixed effect model was used to analyze; otherwise, the random effect model was selected to calculate the pooled effect. Funnel plots were created, and Beggar's test and Egger's regression method were also applied to detect the potential publication bias.

## 3. Results

### 3.1. Search Process

**Figure 1:**
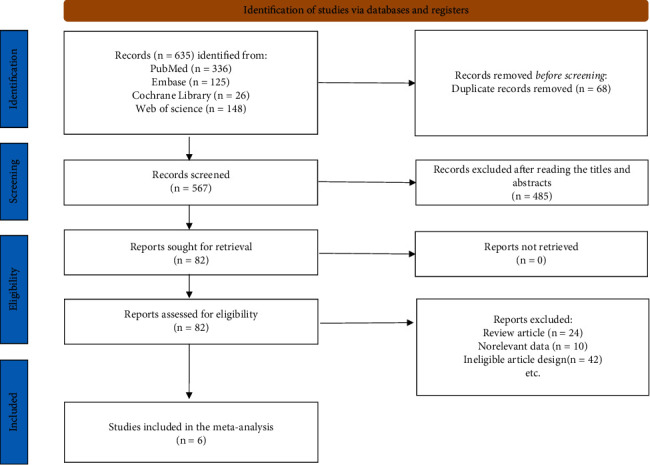
Search strategy used for the inclusion of studies in meta-analysis.

A total of 635 studies were identified following the electronic search. After eliminating duplicate literature searches, 567 studies were screened for titles and abstracts, and 82 studies were considered potentially suitable for inclusion. Following the full paper review, 6 articles met the final criteria for inclusion [15–20]. The search process and full inclusion/exclusion criteria are shown in Figure 1.

### 3.2. Characteristics of Included Studies

Table 2 presents a comprehensive description of each included article in our study. These articles contained six RCTs, all of which were phase III International studies. Novel androgen receptor antagonists included abiraterone (*n* = 2), enzalutamide (*n* = 2), apalutamide (*n* = 1), and darolutamide (*n* = 2). These studies compromised 7748 patients, including 3870 in the test group and 3878 in the control group.

### 3.3. Results of Quality Assessment

**Figure 2:**
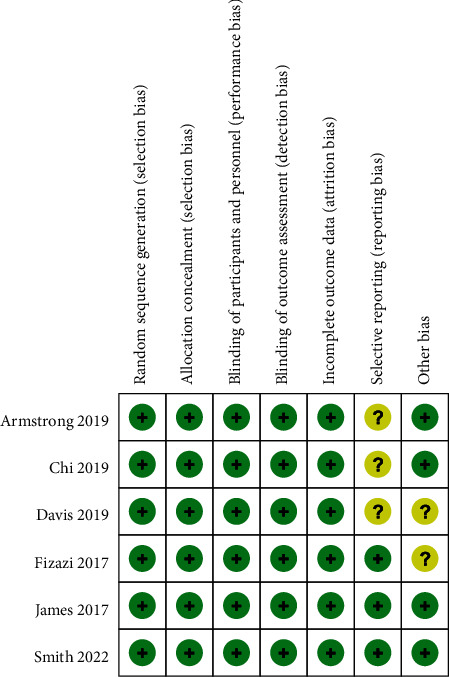
Risk-of-bias assessment of each included studies.

According to the evaluation criteria of the Cochrane Collaboration tool, we made a subjective quality evaluation after a careful reading of the full text. For the included studies, each study generated a random sequence for grouping and used the blinding method. Intention to treat was not used in any studies. Three studies provided fewer outcome variables, and two studies lacked the description of basic information of some patients.  Figure 2 summarizes the risk of bias for each included study.

### 3.4. Meta-Analysis of Survival Outcomes

The summarization of the OS and PFS of all the included studies are presented in Table 3. Four articles reported that the OS rate in the test group was higher than that in the control group. Except that the HR value of the OS in ARCHES was not statistically significant, and the CI of the HR of the other five articles was less than 1, indicating that the therapy in the test group was conducive to improve the OS rate. Three articles reported the median PFS time, and the results in the test group were also higher than those in the control group. The HR values of the PFS in all six studies suggested that the therapy in the test group was helpful to improve PFS.

#### 3.4.1. OS

**Figure 3:**
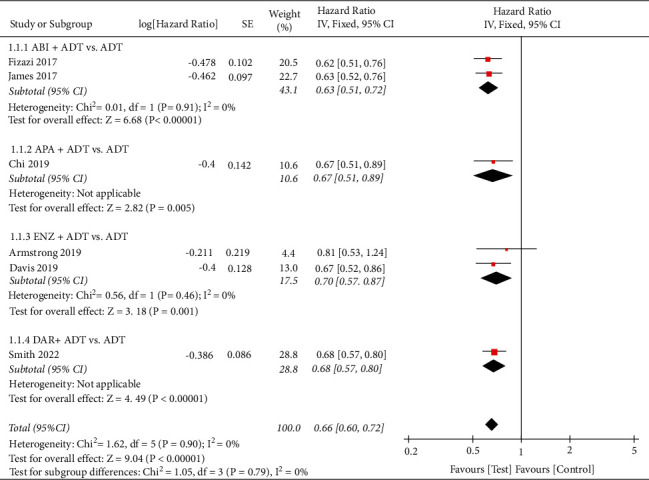
Forest plot: comparison of the overall survival rate. ABI, abiraterone; ADT, androgen-deprivation therapy; ENZ, enzalutamide; APA, apalutamide; DAR, darolutamide.

We performed a meta-analysis to calculate the overall OS rate using the fixed effect model based on heterogeneity analysis (I^2^ = 0%). The pooled HR was 0.66 with 95%CI (0.60, 0.72), while the *P* value of the overall effect was <0.00001 (Figure 3), which indicated that the test group could significantly improve the OS rate compared with the control group.

#### 3.4.2. PFS

**Figure 4:**
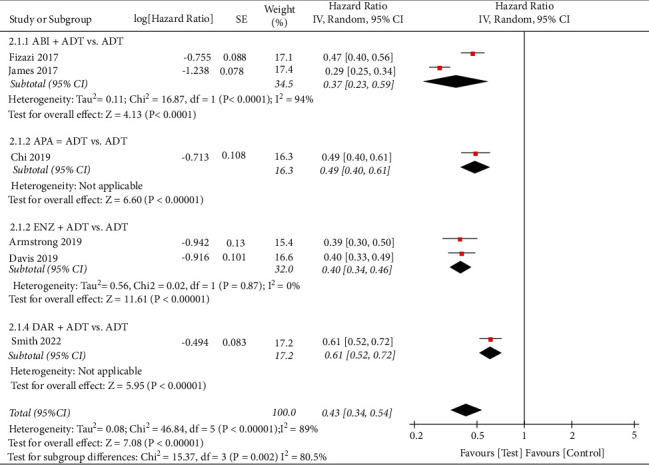
Forest plot: comparison of progression-free survival. ABI, abiraterone; ADT, androgen-deprivation therapy; ENZ, enzalutamide; APA, apalutamide; DAR, darolutamide.

A similar analysis was conducted to evaluate the overall PFS, and the result showed that the test group could also significantly improve the PFS than the control group (HR: 0.43, 95%CI (0.34, 0.54), *P* < 0.00001, fixed effect model) (Figure 4).

### 3.5. Meta-Analysis of Safety Outcomes

AEs were used to reflect the safety of the combination of novel androgen receptor antagonists and ADT. The summarization of any AE, grade 3 or 4 AE, any serious AEs, and AEs leading to death of all the included studies is presented in Table 4. The rates of any AE, grade 3 or 4 AE, any serious AEs, and AEs leading to death in the test group and the control group were 96.1% vs 95.6%, 37.1% vs. 31.5%, 28.1% vs. 25.2%, 3.4% vs. 3.2%, and 96.1% vs. 95.6%, respectively.

#### 3.5.1. AEs

**Figure 5:**
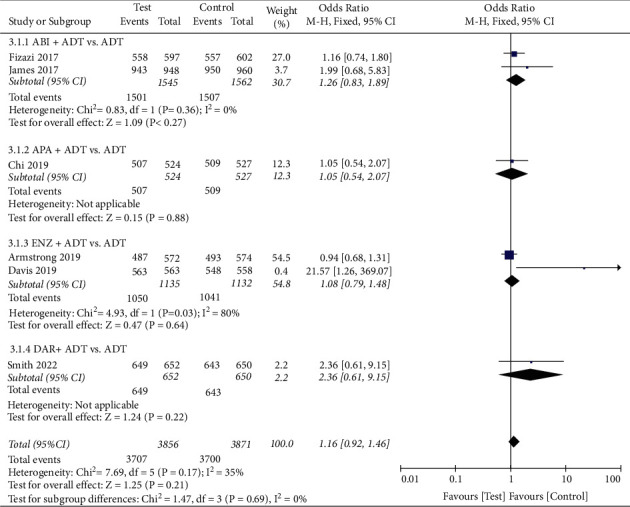
Forest plot: comparison of any adverse event. ABI, abiraterone; ADT, androgen-deprivation therapy; ENZ, enzalutamide; APA, apalutamide; DAR, darolutamide.

**Figure 6:**
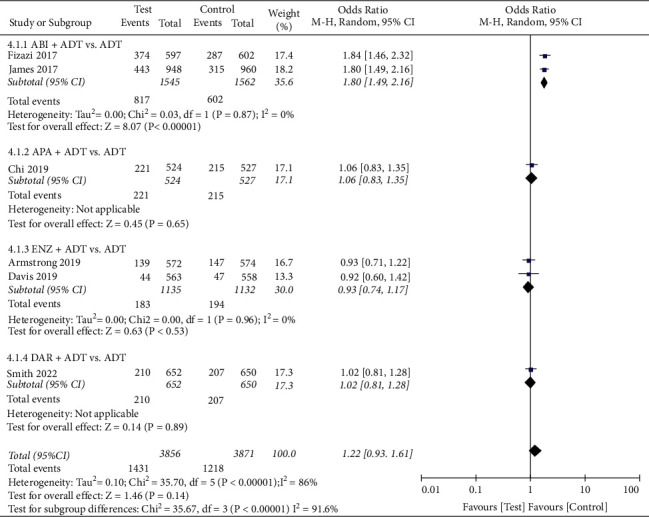
Forest plot: comparison of grade 3 or 4 adverse event. ABI, abiraterone; ADT, androgen-deprivation therapy; ENZ, enzalutamide; APA, apalutamide; DAR, darolutamide.

**Figure 7:**
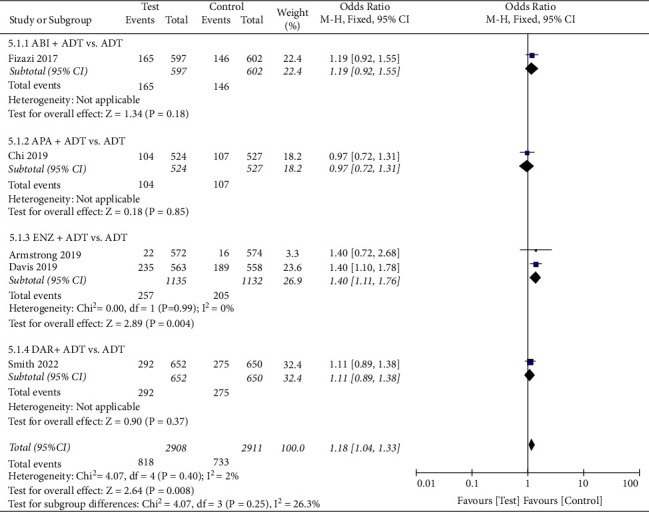
Forest plot: comparison of any serious adverse events. ABI, abiraterone; ADT, androgen-deprivation therapy; ENZ, enzalutamide; APA, apalutamide; DAR, darolutamide.

**Figure 8:**
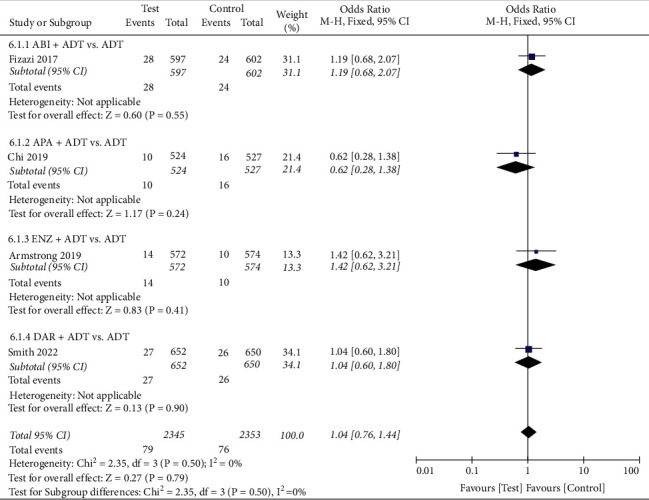
Forest plot: comparison of the adverse events leading to death. ABI, abiraterone; ADT, androgen-deprivation therapy; ENZ, enzalutamide; APA, apalutamide; DAR, darolutamide.

We conducted a pooled analysis of the four types of AEs (Figures 5–8). The results showed that the any serious AE rate of the test group was significantly higher than that of the control group (OR: 1.18, 95%CI (1.04, 1.33), *P*=0.008, fixed effect model, Figure 7), However, there were no statistical difference in any AE rate (OR: 1.16, 95%CI (0.92, 1.46), *P*=0.21, fixed effect model, Figure 5), grade 3 or 4 AE rate (OR: 1.22, 95%CI (0.93, 1.61), *P*=0.14, random effect model, Figure 6), and AEs leading to death rate (OR: 1.04, 95%CI (0.76, 1.44), *P*=0.79, fixed effect model, Figure 5) between the two groups.

### 3.6. Publication Bias

**Figure 9:**
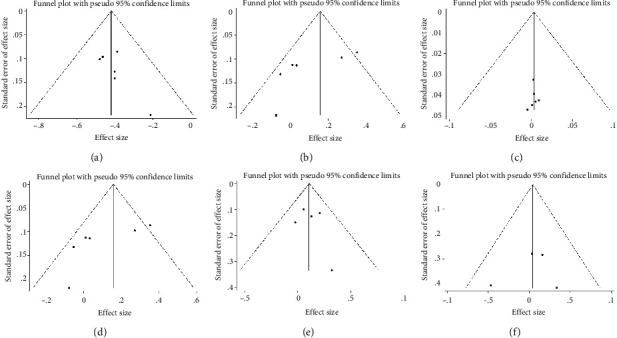
Funnel plot of publication bias risk. (a) Overall survival rate, (b) progression-free survival, (c) any adverse event, (d) grade 3 or 4 adverse event, (e) any serious adverse events, and (f) adverse events leading to death.

Funnel plot analysis for the outcomes of OS, PFS, any AE, grade 3 or 4 AE, any serious AEs, and AEs leading to death was performed to explore the publication bias, although the funnel plots seemed to be asymmetric (Figure 9), neither Beggar's test nor Egger's test showed any publication bias (Table 5).

## 4. Discussion

ADT is the cornerstone of mHSPC treatment. The commonly used methods include surgical castration and drug castration. Drug castration can be divided into gonadotropin releasing hormone agonists and antagonists according to different mechanisms of action. Recent studies have confirmed that the ADT treatment, combined with chemotherapy or new androgen receptor targeting agents, or combined with local treatment of primary/metastatic lesions, can prolong the OS rate and can improve the quality of life of patients [21–23]. ADT alone can only block the production of androgens from the testis but cannot inhibit the secretion of androgens and their derivatives from the pituitary adrenal axis, nor can it block androgens produced by prostate tumor cells; however, ADT combined with novel androgen receptor antagonists can block androgens from multiple sources to a greater extent.

In this study, a meta-analysis was conducted on the efficacy of ADT combined with novel androgen receptor antagonists compared with ADT alone in the treatment of mHSPC, and 6 RCTs were included. In terms of survival outcomes, compared with ADT alone, ADT combined with novel androgen receptor antagonists can significantly improve the OS rate and PFS, which proved the survival benefit of ADT combined with novel androgen receptor antagonists. In terms of safety outcomes, the combination of novel androgen receptor antagonists and ADT would increase the rate of any serious AEs, while in other indicators such as any AE, grade 3 or 4 AE, and AEs leading to death, there was no statistical significance, which proved that the incidence of toxic effects of ADT combined with novel androgen receptor antagonists and ADT alone was similar.

Wang [24] conducted a meta-analysis on the RCTs of ADT combined with abiraterone acetate/enzalutamide/apalutamide in the treatment of mHSPC. The main observation indicators included the OS, PFS, the occurrence of total AEs, and grade 3 or 4 AE. The results showed that ADT combined with novel androgen receptor antagonists could significantly improve the efficacy of mHSPC patients compared with ADT alone, but it can also increase the risk of AEs such as joint pain. Rydzewska [25] conducted a meta-analysis on the treatment of mHSPC with ADT plus abiraterone acetate, although only two studies were included, the results showed that adding abiraterone acetate to ADT was a clinically effective treatment option for mHSPC. Sweeney [26] conducted a meta-analysis on the treatment of mHSPC with ADT plus enzalutamide, and the report suggested that the survival rate of men with cancer recurrence has been improved after adding enzalutamide to ADT. Our study integrated the four currently known novel androgen receptor antagonists (abiraterone/enzalutamide/apalutamide/darolutamide) and combined the clinical outcomes, and our results were similar to the above meta-analysis results.

Abiraterone acetate is an androgen biosynthesis inhibitor, which can highly specifically and irreversibly bind to the rate limiting enzymes CYP17A1, C17 lyase, and C20 lyase for androgen synthesis, thereby inhibiting the androgen synthesis in testicular, adrenal, and prostate tumors [27]. Enzalutamide, a second-generation androgen receptor antagonist, can block the nuclear transport of androgen receptors and the binding of androgen receptors to DNA, thus inhibiting the proliferation of PCa cells and inducing apoptosis. The drug binding affinity of enzalutamide was 5 ∼ 8 times higher than that of the first-generation bicalutamide [28]. Apalutamide is an improved androgen receptor antagonist, and its affinity with an androgen receptor is 7 ∼ 10 times higher than that of kallukamine. Under the condition of androgen receptor over expressions and lack of androgens, apalutamide will not change from an antagonist to an agonist and has stronger antitumor activity [29]. Darolutamide is a new oral next-generation androgen receptor antagonist, which can inhibit the drug resistance of androgen receptor mutants. Due to its low blood-brain barrier permeability, it has a high safety and drug resistance and can reduce the risk of epileptic symptoms. With the development of new drug research and development, more and more novel androgen receptor antagonists will be used in combination therapy in the future [22].

This study had some limitations. Firstly, although the included studies were all multicenter, large sample, and high-quality RCTs, the studies focused on a single novel androgen receptor antagonist were relatively few, which limit the subgroup analysis of a single drug. Secondly, the studies included in the analysis were all carried out in the recent 5 years, and the long-term effect, especially the long-term recurrence rate and long-term cancer metastasis, has not been observed. In addition, the baseline situation of the patients in each study was different, which may lead to some heterogeneity among studies, and reduce the overall quality of the study.

## 5. Conclusions

In conclusion, ADT combined with novel androgen receptor antagonists is effective in mHSPC patients, which can effectively improve the survival status of patients, but the risk of serious AEs also increases. Patients should strengthen the monitoring of adverse reactions while receiving the novel androgen receptor antagonist to avoid serious AEs as much as possible. In clinical practice, combined with the treatment conditions that can be met in various regions, different combined treatment schemes can be selected on the basis of ADT treatment according to the actual needs and physical conditions of patients.

## Figures and Tables

**Table 1:** Search strategies for each database.

Search number	Queries
Queries in pubmed	
#1	Search “metastatic prostate cancer” (mesh)
#2	Search ((metastatic prostate cancer (title/abstract)) or metastatic pancreatic cancer (title/abstract))
#3	#1 or #2
#4	Search (((((androgen-deprivation therapy (title/abstract)) or abiraterone (title/abstract)) or enzalutamide (title/abstract)) or apalutamide (title/abstract)) or darolutamide (title/abstract))
#5	#3 and #4

Queries in cochrane	
#1	MeSH descriptor: (metastatic prostate cancer) explode all trees
#2	((Metastatic prostate cancer^*∗*^) or (metastatic pancreatic cancer^*∗*^)): Ti, ab, kw
#3	#1 or #2
#4	MeSH descriptor: (androgen-deprivation therapy) explode all trees
#5	((Androgen-deprivation therapy^*∗*^) or (abiraterone^*∗*^) or (enzalutamide^*∗*^) or (apalutamide^*∗*^) or (darolutamide^*∗*^)): Ti, ab, kw
#6	#4 or #5
#7	#3 and #6

Queries in embase	
#1	‘Metastatic prostate cancer'/exp or ‘metastatic pancreatic cancer': Ti, ab
#2	‘Androgen-deprivation therapy': Ti, ab or ‘abiraterone': ti, ab or ‘enzalutamide': Ti, ab or ‘apalutamide': Ti, ab or ‘darolutamide': Ti, ab
#3	#1 and #2

Queries in WOS	
#1	TS = (metastatic prostate cancer or metastatic pancreatic cancer)
#2	TI = (androgen-deprivation therapy or abiraterone or enzalutamide or apalutamide or darolutamide)
#3	#1 and #2

MeSH, Medical Subject Headings.

**Table 2:** Characteristics of studies included in this meta-analysis.

Study	Country	Phase	Trial name	Treatments		No. of patients		Age (years)^*∗*^		Median PSA (ng) (ml)^*∗*^	
				Test	Control	Test	Control	Test	Control	Test	Control
James [15]	International	III	STAMPEDE	ABI + PRE + ADT	Placebos + ADT	960	957	67 (42–85)	67 (39–84)	51 (0–21460)	56 (0–10530)
Fizazi [16]	International	III	LATITUDE	ABI + PRE + ADT	Placebos + ADT	597	602	67.3 ± 8.5	66.8 ± 8.7	NR	NR
Chi [17]	International	III	TITAN	APA + ADT	Placebos + ADT	525	527	69 (45–94)	68 (43–90)	5.97 (0–2682)	4.02 (0–2229)
Armstrong [18]	International	III	ARCHES	ENZ + ADT	Placebos + ADT	574	576	70 (46–92)	70 (42–92)	5.4 (0–4823.5)	5.1 (0–19000.0)
Davis [20]	International	III	ENZAMET	ENZ + ADT	ADT	563	562	68.9 ± 8.1	68.8 ± 8.3	NR	NR
Smith [19]	International	III	ARASENS	DAR + DOC + ADT	Placebos + DOC + ADT	651	654	67 (41–89)	67 (42–86)	30.3 (0–9219)	24.2 (0–11947)

PSA, prostate-specific antigen; ABI, abiraterone; PRE, prednisolone; ADT, androgen-deprivation therapy; ENZ, enzalutamide; APA, apalutamide; DAR, darolutamide; DOC, docetaxel; NR, not reported. The symbol ^*∗*^indicates that the data were presented as the median (range) or mean ± standard deviation.

**Table 3:** Survival outcomes of all the included studies.

Trial name	Test vs. control	OS				Median PFS			
		Test (%)	Control (%)	HR (95% CI)	*P* value	Test (mo)	Control (mo)	HR (95% CI)	*P* value
STAMPEDE	ABI + PRE + ADT vs. Placebos + ADT	83	73	0.63 (0.52–0.76)	<0.001	43.9	30.0	0.29 (0.25–0.34)	<0.001
LATITUDE	ABI + PRE + ADT vs. Placebos + ADT	66	49	0.62 (0.51–0.76)	<0.001	33.0	14.8	0.47 (0.39–0.55)	<0.001
TITAN	APA + ADT vs. Placebos + ADT	82.4	73.5	0.67 (0.51–0.89)	0.005	NR	NR	0.49 (0.40–0.61)	NR
ARCHES	ENZ + ADT vs. Placebos + ADT	NR	NR	0.81 (0.53–1.25)	0.336	NR	19.0	0.39 (0.30–0.50)	<0.001
ENZAMET	ENZ + ADT vs. ADT	NR	NR	0.67 (0.52–0.86)	0.002	NR	NR	0.40 (0.33–0.49)	<0.001
ARASENS	DAR + DOC + ADT vs. Placebos + DOC + ADT	62.7	50.4	0.68 (0.57–0.80)	<0.001	51.2	39.7	0.61 (0.52–0.72)	<0.001

OS, overall survival; PFS, progression-free survival; ABI, abiraterone; PRE, prednisolone; ADT, androgen-deprivation therapy; ENZ, enzalutamide; APA, apalutamide; DAR, darolutamide; DOC, docetaxel; NR, not reported.

**Table 4:** Safety outcomes of all the included studies.

Events	Latitude		Stampede		Arches		Enzamet		Titan		Arasens		sum up	
	Test	Control	Test	Control	Test	Control	Test	Control	Test	Control	Test	Control	Test	Control
Any AE	558/597 (93)	557/602 (93)	943/948 (99)	950/960 (99)	487/572 (85.5)	493/574 (85.9)	563/563 (100)	548/558 (98)	507/524 (96.8)	509/527 (96.6)	649/652 (99.5)	643/650 (98.9)	3707/3856 (96.1)	3700/3871 (95.6)
Grade 3 or 4 AE	374/597 (63)	287/602 (48)	443/948 (47)	315/960 (33)	139/572 (24.3)	147/574 (25.6)	44/563 (8)	47/558 (8)	221/524 (42.2)	215/527 (40.8)	210/652 (32.2)	207/650 (31.8)	1431/3856 (37.1)	1218/3871 (31.5)
Any serious AEs	165/597 (28)	146/602 (24)	NR	NR	22/572 (3.8)	16/574 (2.8)	235/563 (42)	189/558 (34)	104/524 (19.8)	107/527 (20.3)	292/652 (44.8)	275/650 (42.3)	818/2908 (28.1)	733/2911 (25.2)
AEs leading to death	28/597 (5)	24/602 (4)	NR	NR	14/572 (2.4)	10/574 (1.7)	NR	NR	10/524 (1.9)	16/527 (3)	27/652 (4.1)	26/650 (4)	79/2345 (3.4)	76/2353 (3.2)

AE, adverse event; NR, not reported. The data were presented as the median case number/total number (incidence × 100).

**Table 5:** Publication bias by Egger's test and Beggar's test.

Outcomes indicators	No. of studies	*P* of Egger's test	*P* of Beggar's test
OS	6	0.177	0.573
PFS	6	0.865	0.851
Any AE	6	0.703	0.348
Grade 3 or 4 AE	6	0.090	0.188
Any serious AEs	5	0.641	0.892
AEs leading to death	4	0.728	0.497

OS, overall survival; PFS, progression-free survival; AE, adverse event.

## Data Availability

The datasets used and analyzed during the current study are available from the corresponding author upon reasonable request.
